# Three-Dimensional Matrix-Induced Autologous Chondrocytes Implantation for Osteochondral Lesions of the Talus: Midterm Results

**DOI:** 10.1155/2012/942174

**Published:** 2012-04-17

**Authors:** B. Magnan, E. Samaila, M. Bondi, E. Vecchini, G. M. Micheloni, P. Bartolozzi

**Affiliations:** Department of Surgery, Orthopaedic and Traumatology Clinic, G.B. Rossi Hospital, University of Verona, Piazzale L. A. Scuro 10, 37134 Verona, Italy

## Abstract

*Introduction*. We evaluate the midterm results of thirty patients who underwent autologous chondrocytes implantation for talus osteochondral lesions treatment. *Materials and Methods*. From 2002 to 2009, 30 ankles with a mean lesion size of 2,36 cm^2^ were treated. We evaluated patients using American Orthopaedic Foot and Ankle Surgery and Coughlin score, Van Dijk scale, recovering time, and Musculoskeletal Outcomes Data Evaluation and Management System. *Results*. The mean AOFAS score varied from 36.9 to 83.9 at follow-up. Average of Van Dijk scale was 141.1. Coughlin score was excellent/good in 24 patients. MOCART score varied from 6.3 to 3.8. *Discussion*. This matrix is easy to handle conformable to the lesion and apply by arthroscopy. No correlation between MRI imaging and clinical results is found. *Conclusions*. Our results, compared with those reported in literature with other surgical procedures, show no superiority evidence for our technique compared to the others regarding the size of the lesions.

## 1. Introduction

Ankle sprain is the most common trauma in sports, and an osteochondral lesion occurs, in up to 50% of an acute ankle trauma [1, 2]. Every day 1 out of 10000 people undergoes to an ankle injury, and in sports practice, this incidence becomes 5,23 out of 10000 [3–5]. The incidence of these lesions is more frequent in male (70%), with an average age ranging between 20 and 30 years, and is bilateral in 10% of cases [6]. The symptoms are impaired function, limited range of motion, stiffness, ankle pain even after a mild traumatic event, or chronic ankle pain [7, 8].

There are also no traumatic causes of osteochondral talus lesions: chronic instability, endocrine or metabolic factors, joint malalignment, idiopathic avascular necrosis particularly dumping growth age, degenerative joint diseases, systemic vasculopathies, and genetic predisposition [9–16].

Osteochondral lesions were classified by Berndt and Harty [17] in 1959 (X-ray classification), modified by Loomer et al. [18] in 1993 (CT scan classification), but many other classifications have been developed, including the arthroscopic outerbridge classification that is commonly used in clinical practice [19–24]. Only the MRI can assess the cartilage damage and other soft tissue lesions [25–27].

The aim of this study was to evaluate the midterm results of a series of patients who have undergone autologous chondrocytes implantation for the treatment of osteochondral lesions of the talus with MACI (Matrix-induced Autologous Chondrocytes Implantation) technique.

Conservative treatments for osteochondral lesions are different, having the aim to improve symptoms and improving function, leading to spontaneous healing in early stages and in younger subjects, while surgical treatment is indicated in case of failure of conservative treatment. The use of autologous chondrocytes implants with 3-dimensional matrices seems to have allowed a breakthrough in the treatment of cartilage lesion and we propose this technique in lesion types 3 and 4 according to the outerbridge classification [19].

Surgical treatment for painful osteochondral lesion of the ankle is increased over the last years. The aim of surgical treatment is the defect revascularization stimulating the formation of a fibrous scar or the reconstruction of the cartilage layer [20, 28–30].

Different surgical procedures, as described in literature, find their indication depending on the type of lesion, so that the decision-making process requires a careful physical evaluation and an instrumental assessment to determine the right choice [31–33].

## 2. Materials and Methods

This study was approved by the institutional review board.

From 2002 to 2009, 30 ankles in the same amount of patients were treated for osteochondral lesions of the talus.

The inclusion criteria in this study were patients affected by a posttraumatic and non traumatic osteochondritis dissecans, aged more than 16 years old and less than 50, osteochondral lesions sized more than 1.5 cm² and less than 4 cm².

Patients with history of arthritis or previous surgical procedures, overweight patients, “kissing lesions”, patients aged more than 50 years, diabetic or HCV patients, and rheumatologic pathology were excluded.

15 patients were men and 15 women, the age ranging between 17 and 49 years old (average age 28,9) at the time of the procedure; the right ankle was affected in 20 patients, the left in 10 patients.

25 patients had a traumatic events and complained pain and functional impairment, with a lateral instability for chronic ligament injury in 6 cases. 5 patients present non traumatic osteochondritis dissecans.

Preoperatively standard X-rays and an MRI were performed [25–27] and lesions were classified according to the Outerbridge classification [19], appearing to be as type 3 or 4, corresponding to type 2 or 2A according to Giannini's et al. classification [20], with a mean size of 2,36 cm²±0, 49 cm². 18 cases (60%) out of 30 were centromedial dome lesions, 7 cases (23,3%) out of 30 were centrolateral lesions, 3 cases (10%) out of 30 were posteromedial lesions, while 2 lesions (6,7%) were antero medial (Table 1).

We used a MACI (Matrix-induced Autologous Chondrocytes Implantation) Hyaff 11 (Verigen, Leverküsen, Germany) in all patients. These 3-dimensional matrices are composed by resorbable natural collagen derived from bovine tissue that facilitate cells expansion in the cartilage defect. The matrix has two surfaces, one appearing smooth and one rougher: in this latter chondrocytes are seeded.

A single surgeon performed all of the procedures.

**Figure 1:**
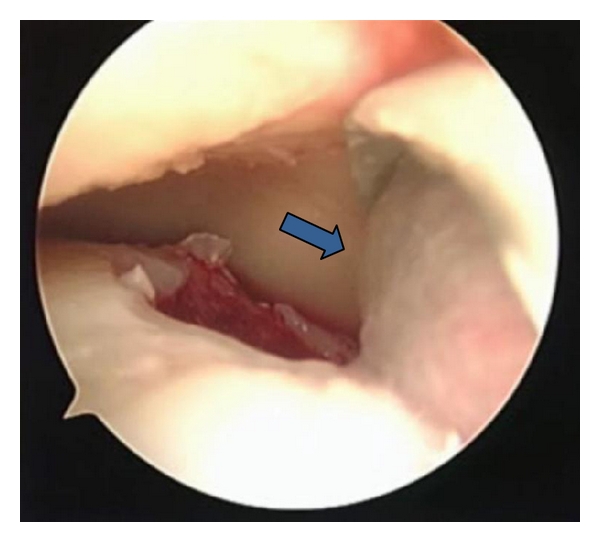
The chondrocytes matrix (arrow) was placed on the cartilage lesion during the 2nd surgical step.

**Figure 2:**
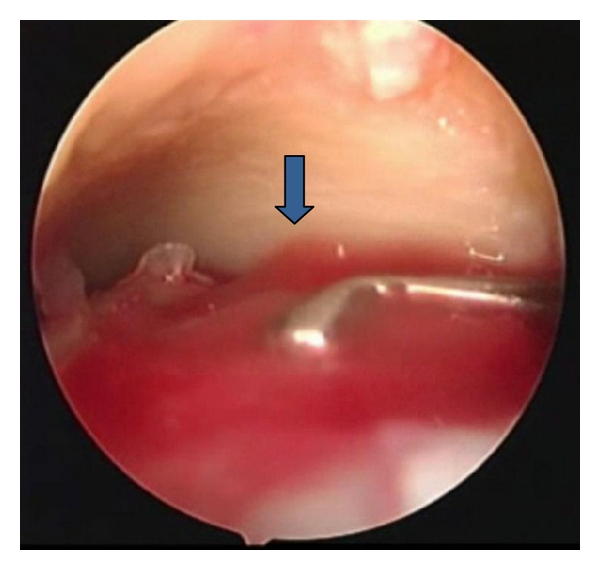
In the 2nd step, after about 30 days, the chondrocytes (arrow) were implanted on the lesion, fixing the matrix with fibrin glue.

The surgical technique was performed in 2 steps, both with spinal anesthesia, in supine position with flexed knee and dangling leg. The 1st step consisted in harvesting a small portion of cartilage arthroscopically from the site of the lesion for chondrocytes culture storing it in vitro with nutrient medium and shipping to Verigen laboratories together with 100 mL of venous patient's blood where chondrocytes development occurs on the 3-dimensional standard size matrix in about 20 days. The 2nd step took place after about 30 days. During this procedure the graft was cut according to the dimension of the template obtained directly on the lesion, implanted and fixed with fibrin glue (Figures 1 and 2). The size of the lesions at MRI was confirmed intraoperatively.

**Figure 3:**
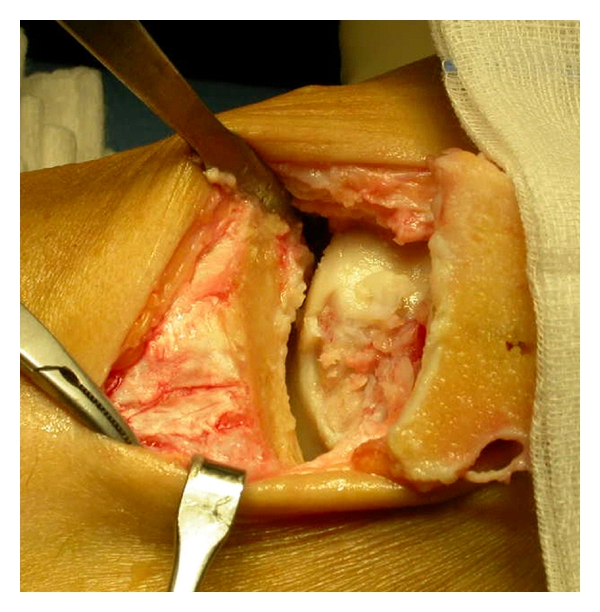
A medial malleolar osteotomy with subsequent fixation of the osteotomy with 1 screw was required in 3 cases to approach in a posteromedial osteochondral lesion of the talus, not suitable by arthroscopy.

**Figure 4:**
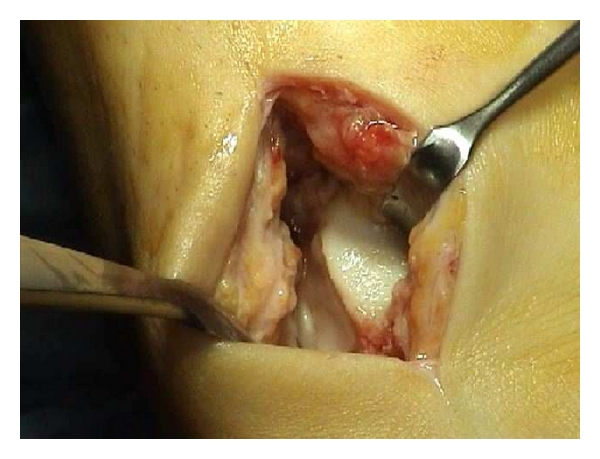
A small arthrotomy was necessary in 2 cases for the achievement of the lesion and the placement of the 3-dimensional matrix with cultured chondrocytes (MACI).

In 25 patients the implant was performed by an arthroscopic approach. Three cases required a medial malleolar osteotomy that was fixed with 1 screw (Figure 3) because of a large posteromedial osteochondral lesion of the talus. In the first 2 cases the lesion was treated by an anterior arthrotomy (Figure 4) [34] because the arthroscopic approach during the learning curve was not successful for the graft implantation.

In 6 ankles (15,4%) out of 30 a lateral ligament reconstruction with a Brostrom-Gould procedure was combined [35, 36]; in addition, in 5 ankles (12,8%) out of 30 a subchondral morcellised bone transplant was performed because of the deep bone degeneration. In the other 2 cases a heel osteotomy to realign the hindfoot and a distraction arthroplasty with external fixation were associated.

Postoperatively patients used crutches without weightbearing for one week after the 1st surgical procedure and for 4 weeks after the second one. A second look for arthroscopic synovectomy was needed in 4 cases for impingement of soft tissue.

We clinically evaluated all patients before surgery and at follow-up using the American Orthopaedic Foot and Ankle Surgery (AOFAS) Ankle-Hindfoot scale score system (rating as excellent the results with a score of more than 80, good with a score range between 61 and 80 points, insufficient when the score was less than 60) [37]. The visual analog scale proposed by Van Dijk et al. (range, 1–190 points; considering positive results when the score was above 114; graded by summing the scores obtained by 19 specific questions) [38], patient's subjective satisfaction according to the method proposed by Coughlin (range, 1–4 points) [39], and information about return to work or sport (range, 0–1 points) were also evaluated at the final follow-up.

Furthermore, for what concerns the MRI, all patients were assessed with the MOCART score (magnetic resonance observation of cartilage repair tissue score) (range, 0–7 points) that is considered to be an index of the graft's integration and its quality [40]. With respect to the osteochondral lesion, it attributes 0 or 1 point for the filling degree, the graft's integration, the lamina's integrity, and for subchondral bone edema; it also assigns 0 to 3 points for MRI signal intensity of the graft's cartilage. It is rated as excellent graft's integration with a score of 0, good with a score range between 1 and 2 points, and sufficient with a score range between 3 and 5 points, insufficient when the score was more than 6.

The preoperative clinical and imaging scores were correlated with the results of the final follow-up using the Student's *t*-test. A *P* value < 0.05 was considered significant. Statistical analysis was performed using Microsoft Office Excel (2007 version).

## 3. Results

The average follow-up was 45 months (range, 18–96 months).

All patients reported reduction or lack of the pain in the ankle area that they had experienced prior to the operation.

**Figure 5:**
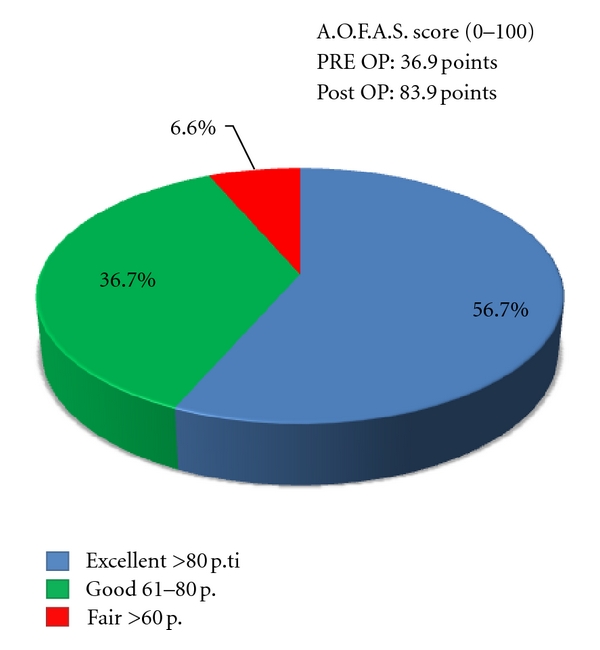
AOFAS score at final follow-up.

The mean total AOFAS score varied from 36,9 ± 6,6 points (range, 30 to 52 points) preoperatively to 83,9 ± 13,6 points (range, 50 to 100 points) at follow-up (*P* < 0,01). We obtain excellent results in 17 patients (56,7%) out of 30, good results in 11 patients (36,7%) out of 30, and insufficient results in 2 cases (6,6%) (Table 2 and Figure 5).

**Figure 6:**
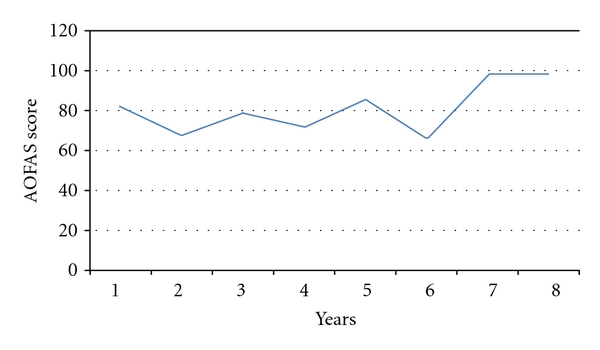
AOFAS score trend during follow-up.

The AOFAS score trend during follow-up remained linear (Figure 6).

The pain score was 40 points of the 40-point maximum on the AOFAS Ankle-Hindfoot scale in 11 patients (36,7%) out of 30, 16 patients (53,3%) out of 30 had occasional mild pain scoring 30 points, while 3 cases (10%) had moderate pain with 20 points.

The functional capacity, which was graded by summing the scores for seven different aspects of functional performance of the scale, averaged 41,9 ± 7,3 points (maximum score on the scale, 50 points), due to the fact that 2 patients (7%) out of 30 complained of ankle-hindfoot instability with activity limitation.

The maximum score for ankle-hindfoot alignment (10 points, indicating excellent or good alignment) was recorded for 24 ankles (80%); a mild, asymptomatic hindfoot malalignment (an AOFAS score of 8 points) was recorded for 5 ankles (16,7%). Only 1 patient had a symptomatic malalignment. The overall mean score for ankle-hindfoot alignment was 9,3 ± 1,9 points.

**Figure 7:**
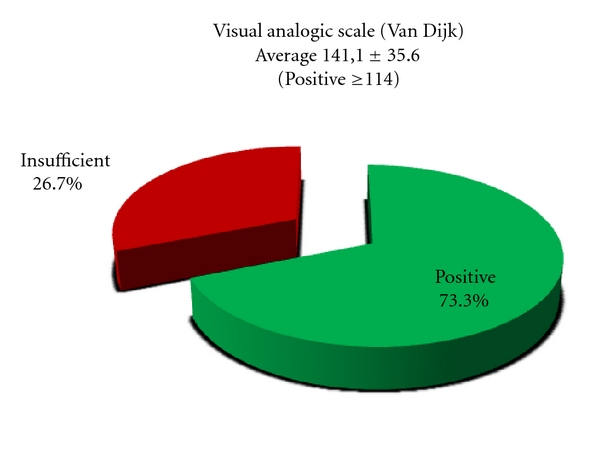
VAS score at final follow-up.

The average of the Van Dijk's Visual Analog Scale (VAS) was 141,1 ± 35,6 points (maximum score on the scale, 190 points) graded by summing the scores for the 19 different questions (Table 1). According to this score we obtained 22 positive results (73,3%) and 8 insufficient results (26,7%) (Table 1 and Figure 7).

**Figure 8:**
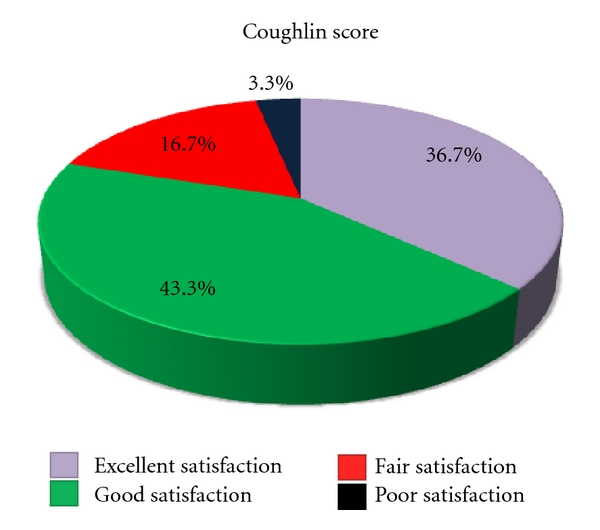
Patient satisfaction Coughlin score at final follow-up.

The patient subjective satisfaction of the patients was sortable as excellent in 11 patients (36,7%), good in 13 (43,3%), fair in 5 patients (16,7%), and insufficient in 1 (3,3%) (Table 1 and Figure 8).

**Figure 9:**
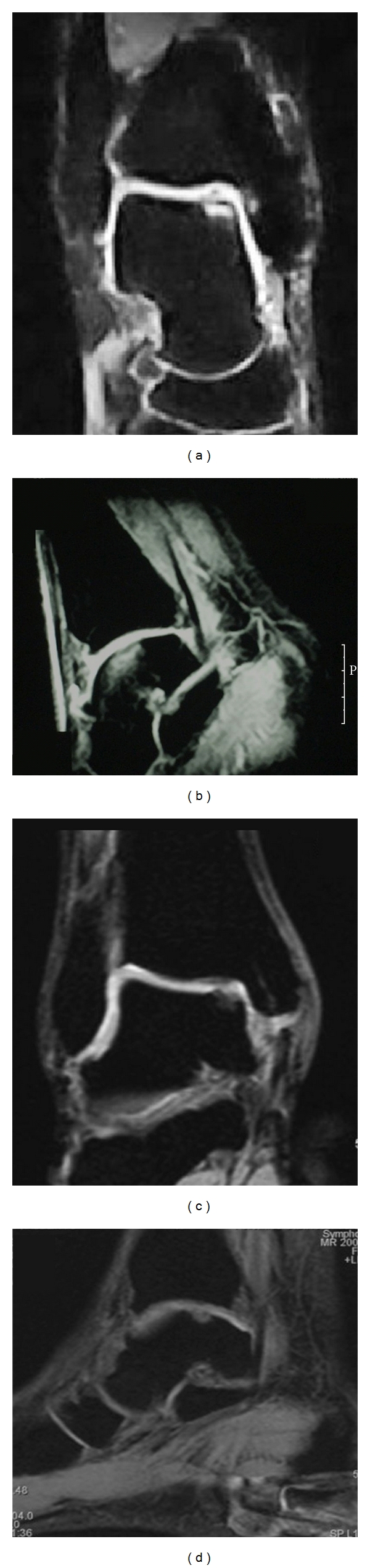
(a, b, c, d) Clinical case: 38-years-old man with anteromedial osteochondral lesion of the talus. Preoperative MRI images (a, b). AOFAS score was 30 points. (c, d) MRI images at follow-up (36 months). AOFAS score was 90 points.

**Figure 10:**
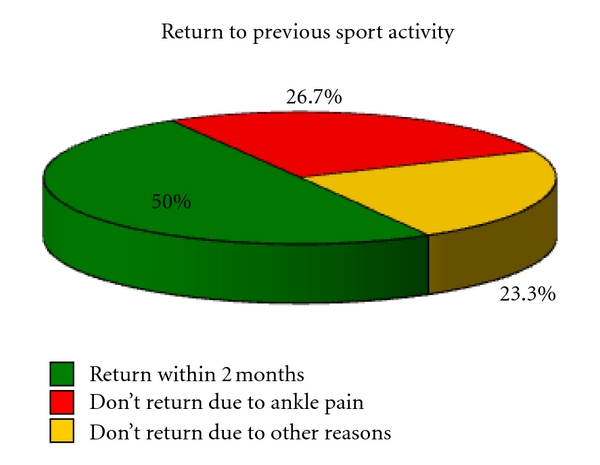
Return to previous sport activity.

With regard to the MRI findings, the preoperatively MOCART score was 6,3 while it became 3,8 at follow-up. This means a good integration of the cartilage graft (Table 1 and Figures 9 and 10).

Fifteen patients (50%) returned to previous sports within 2 months after the surgery, in 8 cases (26,7%) out of 30 sporting activities were not possible for ankle pain, while in 7 patients (23,3%) the no return to sport was not related with clinical outcome (Figure 10).

No major complications as avascular necrosis of the talus or deep infection were observed; 2 anterior impingement and 2 failures of the graft with recurrent lesions occurred in 4 ankles (13,3%) which required an arthroscopic second look. In all these 4 cases the biopsy's histological result was fibrocartilage.

The preoperative and final follow-up AOFAS and MOCART scores were correlated using the Student's *t-*test finding a *P* value nonsignificant.

## 4. Discussion

In literature many authors reported different treatments of the osteochondral lesion [31, 32], but a true comparison between studies is difficult considering the different rating scales used, as indicated by Zengerink et al. in a meta-analysis [33], in which so many scores as AOFAS Ankle-Hindfoot scale, Hannover score, patient satisfaction score, criteria proposed by Berndt and Harty, visual analog scale, Martin score, Alexander and Lichtman, Ogilvie Harris score, MODEMS (Musculoskeletal Outcomes Data Evaluation and Management System), Karlsson scoring scale, Tegner score, evaluation proposed by Loomer, Mazur score, Freiburg ankle score, SANE (Single Assessment Numeric Evaluation), according to Thompson and Loomer, and McCullough score are used for clinical assessment.

Traumatic cartilage fragments that have not detached from the bone can be treated with stabilization by pins or screws [41, 42].

The medial dome lesions are more common and larger than lateral, the two most common talus sites of injury being, respectively, centromedial and centrolateral. Posteromedial and anterolateral lesions are rare [34]. Only 17% of the medial lesions and 20% of the lateral need an osteotomic access, and different studies describe medial malleolar osteotomy access [43–45]. Depending on the treatment methods a precise reduction and fixation of the bone window with 1 or 2 screws is fundamental.

When cartilage remains intact on the subchondral bone lesion, retrograde drilling can be performed to protect the integrity of the articular cartilage. Outcome studies have shown good results [46–48]. Some authors injected, in liquid form, calcium sulfate into the lesion after drilling, as bone substitute [49].

Microfracture or microdrilling has the aim to stimulate the development of fibrocartilage increasing the serum factors on the subchondral plate that leads to fibrous tissue formation at the defect site and symptomatic relief for the patient [50]. The new angiogenesis is by this way stimulated, bone marrow cells are introduced in the osteochondral defect, and fibrocartilaginous tissue is formed. Studies indicate this technique of bone marrow stimulation (BMS) as optimal in small lesion (diameter less than 15 mm) with chondral damage, but not subchondral bone involvement [30, 42, 51, 52].

Tissue transplantation today includes different techniques that are most widely used. Often the surgeons utilize perpendicular access to the injured area to allow the transplant into the talus.

For larger talar injury a Mosaicplasty proposed by Hangody et al. can be performed [53]. The lesion must have a surface of no more than 4 cm² and about 10 mm in diameter. This technique utilizes cylindrical osteochondral plugs taken from the nonweightbearing femoral segment of the knee that are transferred to a talar dome defect. Different studies have shown good and excellent results in 94% of the cases [53–55].

Another technique for larger cystic osteochondral lesion is Osteochondral Autologous Transfer System (OATS). When the lesion is more than 6 mm of diameter, there is the conversion from arthroscopy to open surgery, often with medial malleolar osteotomy. Also in this case good results are reported in literature [56, 57].

Osteochondral Allograft Transplantation is usually utilized for very large lesions, when the size of the lesion is more than 3 cm³. The advantage of this technique is the correct sizing of the graft, which is done intraoperatively with direct measurement. The allograft is often held in place by screw fixation [58–60].

The ACI technique (Autologous Chondrocytes Implantation) was developed by Brittberg et al. and Peterson et al. [61, 62]. It includes the suture of a periosteal flap to the rim of the debrided chondral defect. We can consider MACI technique (Matrix—induced Autologous Chondrocytes Implantation) as an ACI evolution. The advantages of these 3-dimensional matrices are that they are easier to handle and apply, they are conformable to the lesion without the need of periosteal coveraging, and it is possible to apply them by an arthroscopic approach. This is the technique described in this study.

The disadvantages can be found in the need of 2 surgical steps, higher costs, more recovery time, and some limits in the treatment of deep lesions, in which an additional bone graft could be necessary, and some difficulties in new chondrocytes proliferation control [33].

Our results, according to AOFAS score [37], Van Dijk's et al. visual analogic scale (VAS) [38], Coughlin score [39], information about return to work or sport, and the MOCART score [40], are comparable to the results reported in literature [20, 29, 30].

While a preoperative subjective patients' satisfaction Coughlin score cannot be assessed, the lack of the preoperative VAS Van Dijk's score represents a limit of the study.

In the Zengerink et al. review [33] 4 studies with the ACI technique for a total of 59 patients were discussed. In 45 of 59 cases (76%; range: 70 to 92%) a successful result was reported.

Giannini et al. with ACI technique in 8 patients and a mean follow-up of 26 months obtained an increase of the mean AOFAS score from 32,1 preoperatively to 91 postoperatively [28].

According to the literature's results in prospective randomized studies, the ACI technique showed better results in cartilage lesions with a size of more than 3 cm², when compared with mosaicplasty and microfracture technique [63–65].

There are not yet superiority evidence of the MACI technique we applied compared to the ACI technique. This may also be due to assessment methods. Most of the studies use the AOFAS score, which is very “functional” but not specific; it relates to common daily activities [37].

If we judge the results based on the Van Dijk score or on Coughlin's degree of subjective satisfaction, we would see lower excellent and good results.

It is therefore still not possible to adequately compare the two surgical techniques.

Also with MACI technique, as in Brittberg et al. and Peterson et al. technique (ACI) [61, 62], the disadvantage are the 2 surgical steps, in which the 2nd sometimes required a malleolar osteotomy access or anterior arthrotomy, and the cost/benefits ratio of the surgical technique that, according to the different scores used in the studies, cannot be accurately validated.

There are new methods that are still under study.

Mesenchymal Stem Cells (MSCs) from bone marrow have been cultured in vitro and have been used in hybrid scaffolds to repair osteochondral defects [66–68].

Platelet-rich plasma is characterized by red blood cells, white blood cells, and platelets in a fibrin matrix, where platelet-derived growth factor, insulin-like growth factor, and TGF-*β* are included. This clot is poured into the lesion site [69, 70].

Tissue engineering has been proposed for the tissue regeneration using biomaterials, cells, and factors alone or together. Three options have been described: extraction of the patient's cells, vitro cultures, and then transplantation; utilization of biologic factors as growth factors; and use of 3-dimensional porous materials to stimulate the tissue ingrowths [71, 72].

As summarized by Van Dijk et al., ACI/MACI technique is a relatively expensive technique, while OATS gives morbidity from knee complained by up to 36% of patients in literature. On the other hand, arthroscopic excision, curettage, and BMS are relatively inexpensive, with a low morbidity, a quick recovery, and a high success rate (85%).

According to these facts and based on a non-obvious superiority of one surgical technique over the other, BMS should be still considered as the treatment of choice for type 3 and 4 osteochondral talar lesions, while in lesions with a size of more than 1,5 cm², ACI-MACI techniques should be considered in order to achieve better results with cartilage reconstruction.

## 5. Conclusions

Three-dimensional matrices can be considered for single Outerbridge lesions type 3 or 4, sized more than 1,5 cm², patients aged less than 50 years without degenerative changes. Contraindications are overweight patients, “kissing lesions”, patients aged more than 50 years, diabetic or HCV patients, and rheumatologic pathology.

No correlation between MRI imaging and clinical results is found, while histology has shown the formation of hybrid cells between cartilage and fibrocartilage. Probably the marrow edema, generally an inflammation expression, may be caused by a vascular repair hyperflow. Autologous chondrocyte implantation showed consistently better fill of the defects at all times compared with microfracture [73]. Nevertheless MRI remains the gold standard for the instrumental monitoring, according to its high sensibility and tolerability and to its noninvasive and accurate imaging with dedicated scores [63].

The results of this study, however when compared with those reported in literature with other surgical procedures, show that there is not yet a superiority evidence for ACI/MACI technique against microfracture and microdrilling or OATS regardless of the size of the lesions [31–33].

## Figures and Tables

**Table 1:** Patient's data: age, gender, side, and size of lesion.

Age	Gender (M/F)	Side (L/R)	Lesion size (cm²)
33	M	L	1,8
31	M	R	2,4
25	W	R	1,7
29	M	R	2,5
17	M	L	2,1
25	W	R	2,0
21	W	R	2,8
31	W	L	3,7
28	M	R	2,2
22	W	L	1,8
35	M	L	2,6
30	W	R	2,5
29	M	R	1,6
28	W	R	2,5
27	M	L	2,3
19	M	R	2,6
49	W	L	2,8
32	M	R	3,1
41	W	L	2,3
28	M	R	2,7
22	W	R	2,1
38	M	R	2,1
33	M	R	2,5
22	W	R	3,2
24	M	L	1,8
23	W	R	2,2
31	W	L	1,7
25	M	R	1,9
29	W	R	2,5
40	W	R	2,8

**Table 2:** AOFAS, VAS, Coughlin, and MOCART scores' mean values before surgery and at follow-up.

Score	Preop. average ± sd	Follow-up average ± sd
AOFAS (0–100)	36,9 ± 6,6	83,9 ± 13,6
VAS (0–190)		141,1 ± 35,6
COUGHLIN (1–4)		3,1 ± 0,8
MOCART (0–7)	6,3 ± 0,8	3,8 ± 0,9
